# Ploidy status and copy number aberrations in primary glioblastomas defined by integrated analysis of allelic ratios, signal ratios and loss of heterozygosity using 500K SNP Mapping Arrays

**DOI:** 10.1186/1471-2164-9-489

**Published:** 2008-10-17

**Authors:** Paul J Gardina, Ken C Lo, Walter Lee, John K Cowell, Yaron Turpaz

**Affiliations:** 1 Affymetrix, Inc., 3420 Central Expressway, Santa Clara, California 95051, USA; 2 Roche NimbleGen Inc., 500 South Rosa Road, Madison WI 53719, USA; 3 MCG Cancer Center, Medical College of Georgia Medical School, CN 4112, 1120 15^th ^Street, Augusta Georgia 30912, USA; 4 Lilly Singapore Centre for Drug Discovery, 8A Biomedical Grove, #02-05 Immunos, 138648, Singapore

## Abstract

**Background:**

Genomic hybridization platforms, including BAC-CGH and genotyping arrays, have been used to estimate chromosome copy number (CN) in tumor samples by detecting the relative strength of genomic signal. The methods rely on the assumption that the predominant chromosomal background of the samples is diploid, an assumption that is frequently incorrect for tumor samples. In addition to generally greater resolution, an advantage of genotyping arrays over CGH arrays is the ability to detect signals from individual alleles, allowing estimation of loss-of-heterozygosity (LOH) and allelic ratios to enhance the interpretation of copy number alterations. Copy number events associated with LOH potentially have the same genetic consequences as deletions.

**Results:**

We have utilized allelic ratios to detect patterns that are indicative of higher ploidy levels. An integrated analysis using allelic ratios, total signal and LOH indicates that many or most of the chromosomes from 24 glioblastoma tumors are in fact aneuploid. Some putative whole-chromosome losses actually represent trisomy, and many apparent sub-chromosomal losses are in fact relative losses against a triploid or tetraploid background.

**Conclusion:**

These results suggest a re-interpretation of previous findings based only on total signal ratios. One interesting observation is that many single or multiple-copy deletions occur at common putative tumor suppressor sites subsequent to chromosomal duplication; these losses do not necessarily result in LOH, but nonetheless occur in conspicuous patterns. The 500 K Mapping array was also capable of detecting many sub-mega base losses and gains that were overlooked by CGH-BAC arrays, and was superior to CGH-BAC arrays in resolving regions of complex CN variation.

## Background

Changes in chromosomal copy number are common events in tumorigenesis and include homo- or hemizygous deletions, partial or complete duplication of chromosomes, general polyploidy and high copy number amplifications of specific regions. In addition, loss of heterozygosity (LOH) may occur through chromosomal loss or recombination, frequently exposing deleterious recessive genotypes [1]. LOH events can also occur in a copy number neutral manner and would not be detected using copy number analysis only. In total, these changes may correlate with increased expression of oncogenes or inactivation of tumor suppressor genes [2,3]. While many chromosomal alterations may be the result of general karyotype instability in tumor samples, specific regions repeatedly found to be subject to chromosomal copy number aberrations (CNAs) and LOH suggest regions that may carry causative factors in the etiology of the cancer.

Comparative genomic hybridization arrays were developed to probe genomic CNAs in cancer [4,5] as well as other genetic disorders. BAC arrays carry a genomic library as inserts in bacterial artificial chromosomes (BACs), which are spotted onto the array [6]. The arrays are then probed with differentially labeled DNA from reference (normal) and test (tumor) samples, and the relative fluorescence of the two DNAs provides a quantitative estimate of changes in copy numbers within the tumor genome [7]. Increasing densities of the BACs on the arrays generally provide a greater resolution for genomic alterations.

Genomic mapping arrays were developed initially for whole genome genotyping for association studies [8] and later applied to estimate copy number [9-11]. Mapping arrays contain short oligomers to probe the alternative alleles at single nucleotide polymorphisms (SNPs) throughout the genome. Unlike large insert clone arrays, which detect the total signal from relatively large contiguous segments of DNA, mapping arrays provide "point" estimates of the signal intensity at intervals determined by the number and distribution of selected SNPs. The CN value at a particular SNP probe is estimated as a log_2 _ratio (LR) of the total signal from both alleles relative to the signal generated by a "normal" reference population. The expectation is that, in any particular region in the reference population, the CN on average equals 2. Thus, increases or decreases in the LR would reflect gains or losses, respectively, within the genome compared with a diploid reference level. Furthermore, the signals from the alternative alleles can be segregated and translated into specific genotypes on mapping arrays. This allows the identification of LOH by detecting contiguous stretches of homozygous alleles that are statistically unlikely when compared to the heterozygosity frequency of that region relative to the reference samples.

The resolving power of arrays is tied to the size, number and distribution of the probes. The RPCI 6 K BAC array, for example, has a maximum resolution of approximately 500 kb [2] although in practice it is closer to 1 Mbp. The Affymetrix 100 K Mapping array has a mean marker distance of 24 kb with a median of ~8.5 kb. In a study of CNAs involved in mental retardation, deletions as small as 178 kb were detected using the 100 K platform. These deletions were undetectable using conventional cytogenetic methods [12]. In a parallel study of gliomas, the 100 K platform was better able to resolve chromosomal breakpoints and could detect novel homozygous deletions as small as 50 kb [13] compared with a 6 K BAC array. Because the Affymetrix 500 K Mapping array used in the current study has an average inter-SNP distance of 5.8 kb, it was expected to demonstrate a nearly proportional increase in resolution.

Mapping arrays were developed to assay the genotype of predominantly diploid samples, and CN analysis detects regions of generally increased or decreased total signal against an assumed diploid background. Tumor samples may violate this assumption, frequently displaying genomes that are largely polyploid due to large scale aberrations in the mitotic mechanism, such as endoreduplication. The primary restriction for accurate ploidy analysis in this case is probably the experimental protocol, which calls for a standard amount of DNA to be assayed. Such procedures will approximately equalize the total signal regardless of whether the original cells were diploid or, for example, tetraploid. In addition, analytical procedures tend to normalize the overall signal approximate to those found in the reference population, which is almost exclusively diploid. As a result, computational methods relying on the total DNA signal will tend to grossly underestimate the actual copy number of tetraploid samples, since the presumptive baseline signal is set at two rather than four.

The capacity of mapping arrays to detect signals from individual alleles also permits the calculation of an allelic ratio (AR) – the relative contribution from a given allele to the overall signal [14]. The AR generates patterns that are characteristic of particular classes of CNAs, and these patterns can be distinguished from that of heterozygous diploidy. Previous work [13] reported a discrepancy between CN estimations and LOH predictions using a 100 K mapping array analysis of glioblastoma multiforme (GBM) tumors. This observation led to the suggestion that many of the analyzed chromosomes might have a baseline CN greater than two. Here, we perform an integrated analysis of GBM tumors utilizing log ratios (LR), LOH predictions and allelic ratios (AR) to demonstrate that, in fact, the chromosomal CNs are frequently much higher than expected from the signal ratios, and that many or all of the chromosomes in particular samples are aneuploid.

## Methods

### Processing of samples for GeneChip^® ^500 K Mapping Arrays

The glioma samples used in this study were collected with informed consent for research purposes and DNA was prepared from snap frozen tissue using standard procedures. In this cohort, all 24 samples were confirmed as GBM by histopathological analysis. HapMap reference samples were obtained from Coriell (Camden, New Jersey, USA) and carry a broad consent for use in genetic variation research.

Labeled DNA target was prepared according to the Affymetrix Mapping 500 K protocol. Two separate reactions, each containing 250 ng of genomic DNA, were digested with either NSP I or STY I (NEB, Ipswich, MA) at 37°C for 2 hours, then 65°C for 20 minutes. Corresponding NSP I and STY I Adaptors (Affymetrix Inc., Santa Clara, CA), which consist of a universal PCR primer sequence, were ligated using T4 DNA Ligase (NEB) at 37°C for 3 hours, then 65°C for 20 minutes. The reactions were PCR-amplified in triplicate, using TITANIUM Taq (Clontech, Mountain View, CA) and PCR Primer 002 (Affymetrix).

An Applied Biosystems 9700 instrument was used with the following cycling parameters: one 3 min 94°C polymerase activation step, followed by 30 cycles of three-step PCR (15 s of 94°C denaturation, 45 s of 60°C annealing, and 15 s of 68°C extension), and a final 68°C, 7 min extension. 8 uL 0.1 M EDTA was added to each PCR product and the triplicate amplification reactions were pooled into a Clean-Up Plate (Clontech), washed 3 times with water, and eluted with 50 uL Recovery Buffer (Clontech).

The purified PCR product was quantitated using a Molecular Devices SpectraMax Plus 384 Plate Reader. 90 μg of PCR product was fragmented with Fragmentation Reagent (Affymetrix) at a final reaction concentration of 0.005 U/μL. A 9700 instrument was used to fragment at 37°C for 35 minutes, then 95°C for 15 min. Labeling of target used 30 mM DNA Labeling Reagent, 30 U/μL Terminal Deoxynucleotidyl Transferase, and 5× TdT Buffer (Affymetrix). Labeling was performed at 37°C for 4 hours, followed by 95°C for 15 min.

190 μL of Hybridization mixture was added to each sample as outlined in the Mapping 500 K protocol. The labeled target was heat-denatured at 95°C for 10 min, then 49°C for a minimum of 5 minutes prior to adding 200 μl of target to a corresponding Mapping 250 K NSP or STY Array. The array was allowed to incubate in a rotating hybridization oven at 49°C, 60 RPM, for 16 hours. The target was removed from the array and subsequently stained and washed on an Affymetrix Fluidics 450 Station using washing protocol Mapping500Kv1_450. The arrays were scanned using an Affymetrix 7G GeneChip Scanner and GCOS Version 1.4 software.

### Analysis of 500 K genotyping arrays

Allele signal summaries and genotypes were generated from CEL files with the command-line program apt-probeset-genotype (v. 1.6.0; the Affymetrix Power Tool [APT] package is available at [15]). The files were processed using the Bayesian Robust Linear Model with Mahalanobis distance (BRLMM) algorithm implemented in APT [16] with a quantile sketch normalization of 50,000 points and no background correction. 500 K arrays from 51 female samples from the International Hap Map project were used in the normalization and as baseline reference signals for CN estimations. Copy number and LOH were estimated with the apt-copynumber-pipeline program [17] with Gaussian smoothing at 0.1 Mb. The Nsp and Sty arrays are processed separately by apt-probeset-genotype but integrated by the copynumber-pipeline program. These basic functionalities are also available in the CNAT4 software [18]. Copy number is estimated as a log-sum of the normalized sample allele signals (S) against those of the reference set (R):

Log Ratio = log_2 _(S_a_/(R_a_+R_b_) + S_b_/(R_a_+R_b_))

where a and b refer to the alternative SNP alleles.

LOH is estimated from the sample genotypes by a Hidden Markov Model using the SNP-wise heterozygosity rate of the reference population and a genotyping error of 0.02 (default) to estimate the prior. The transition decay parameter (describing the influence of the LOH state of neighboring SNPs) was set to 10 Mb. The state prediction of "LOH" versus "retention of heterozygosity" (1 or 0) by the HMM was used to map regions of empirical LOH. The CN and LOH algorithms are described in [19].

The allelic ratio represents the ratio of the B allele intensity to the total A allele plus B allele intensity. The approach is based on that described in Peiffer et al. [14], in which the ratio from each particular SNP was normalized by linear interpolation against a set of reference samples to account for variation in individual SNP cluster characteristics. Allelic ratios were calculated with the Partek Genomics Suite, version 6.3 (Copyright^© ^2008, Partek Inc., St. Louis, MO, USA) using 270 HapMap samples for normalization and a proprietary noise reduction algorithm. Diploid homozygotes are expected to have ratios near 0 or 1, and diploid heterozygotes are expected to have ratios near 0.5 (i.e., an equal contribution by both alleles). Log ratios, allelic ratios and LOH predictions were visualized in a genomic context within the Partek^® ^Genomics Suite.

For CN estimations from mapping arrays, the Inter-Quartile Range (IQR) is used as the basic QC metric. IQR is a measure of the variability of the signal across the genome of an individual sample and, in a relatively normal sample, will reflect the general noisiness of the sample. However, given the frequency and extent of CN aberrations in tumor samples, standard IQR metrics are only a rough guide of sample noise.

## Results

The chromosome complement in a tumor sample can profoundly affect the interpretation of losses and gains within the karyotype, and conventional CGH using comparative hybridization between tumor and normal samples does not evaluate this variable. The SNP mapping arrays, however, can establish hybridization intensities at each allele, and interpretation of the relative intensity patterns along the length of the chromosome reveals valuable information about the ploidy of a tumor sample. To study this approach further we have analyzed a series of 24 glioma samples using the 500 K SNP mapping arrays. In all of the figures supporting this study, we have adopted the convention of the short arm of the chromosome being placed to the left and the long arm to the right. To aid in the interpretation of the figures, we have included a red line tracing the general median point of equivalent segments of the allelic ratios.

### Patterns of allelic ratios

**Figure 1:**
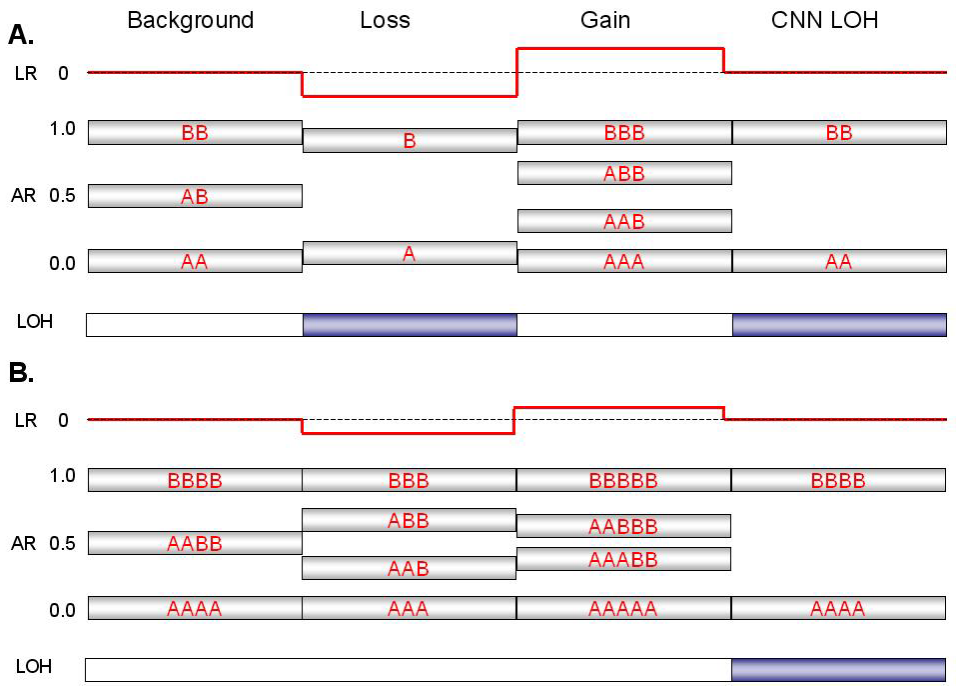
**Expected signal patterns for chromosomal changes against a background of (A) diploidy or (B) tetraploidy**. The cartoon summarizes the patterns of log ratio, allelic ratio and LOH that would accompany events (a single-copy gain, a single-copy loss, or copy-neutral LOH) at four hypothetical segments of either a disomic chromosome in a diploid background (Panel A) or tetrasomic chromosome in a tetraploid background (Panel B). A log ratio (red line) of 0 indicates that copy number (CN) is unchanged relative to the baseline, which equals 2 for a normal diploid sample. The allelic ratio is the proportion of total signal generated by the B allele probe (e.g., a genotype of AAB at a particular SNP produces an allelic ratio of 0.33 [B/A+A+B]). Similar ratios from many contiguous SNPs are shown as silver boxes with red letters (various combinations of A and B) indicating the inferred genotype that is responsible for the AR value. Segments expected to produce LOH are indicated by blue boxes. Note that for a balanced tetrasomic chromosome in a tetraploid sample (Panel B), the Background state is indistinguishable from diploidy (Panel A); the LR of 0 reflects the baseline copy number of the sample, which equals 4. The two cases are only distinguishable at losses or gains, which alter the pattern in divergent ways.

**Figure 2:**
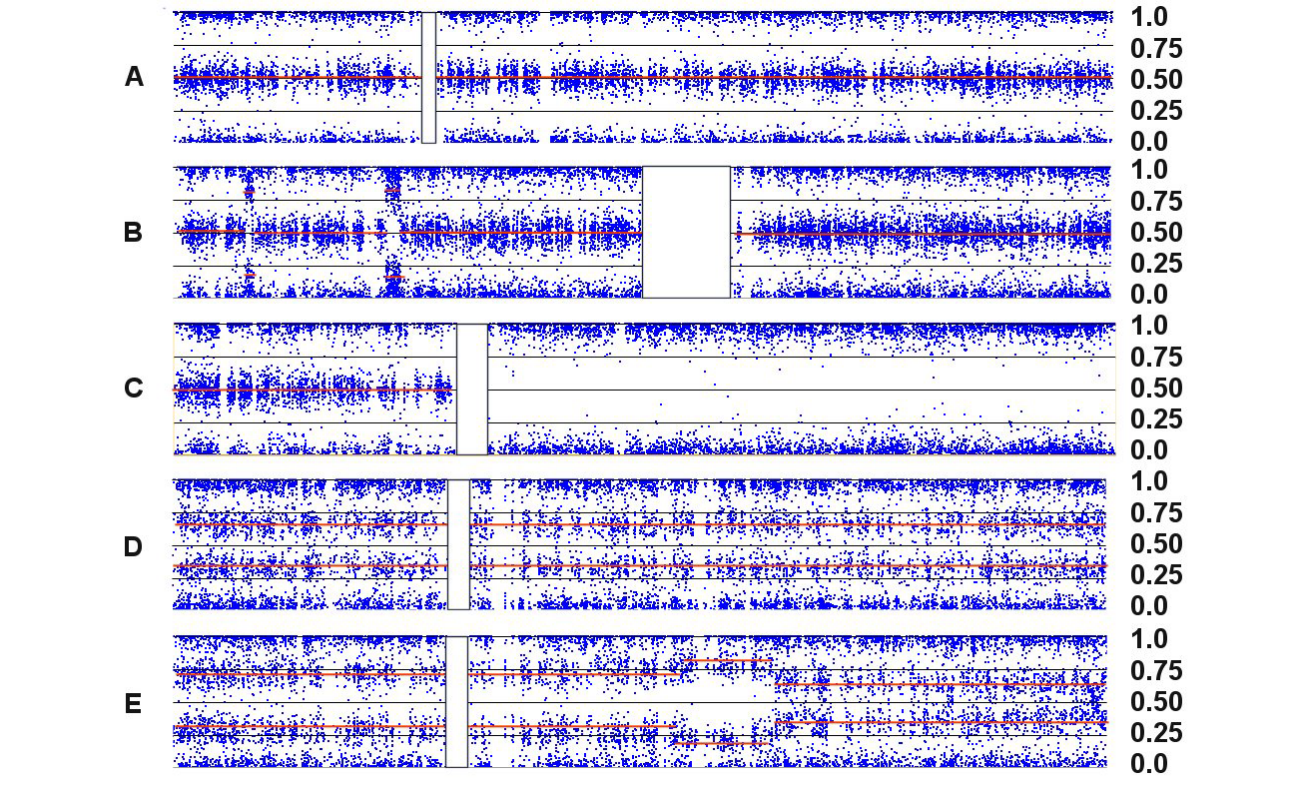
**Patterns of Allelic Ratios (AR)**. Each point (blue dot) represents the AR for one SNP mapped to its relative physical position along the length of the chromosome from the p-terminus (left) to the q-terminus (right). The vertical white rectangles indicate the position of the centromeres, which lack SNP probes. (A) In Chr:5 from sample C92, three 'bands' are evident in a disomic chromosome; ARs of 0 and 1 represent homozygous signals (AA or BB) while an AR of ~0.5 represents an equal contribution from both alleles (AB). (B) Two small deletions in the p-arm of disomic Chr:1 in C79 produce characteristic patterns that lack heterozygote signal and show some bleeding of the single-copy homozygotic signal (one A or B allele) toward the middle. (C) The copy-number neutral LOH seen on the long arm of Chr:8 in C156 also lacks heterozygous signal, but does not show inward bleeding. (D) A trisomic pattern for Chr:10 in C82 is characterized by heterozygotic ratios near 0.33 (AAB) and 0.67 (ABB), rather than at 0.5. (E) An unbalanced tetrasomy pattern for Chr:10 in C72, reflecting heterozygote ratios of 0.25 (AAAB) and 0.75 (ABBB), is seen on the short arm, but the ARs shift to an unbalanced pentasomic (ABBBB) and then a trisomic pattern (ABB) near the mid-point of the long arm.

**Figure 3:**
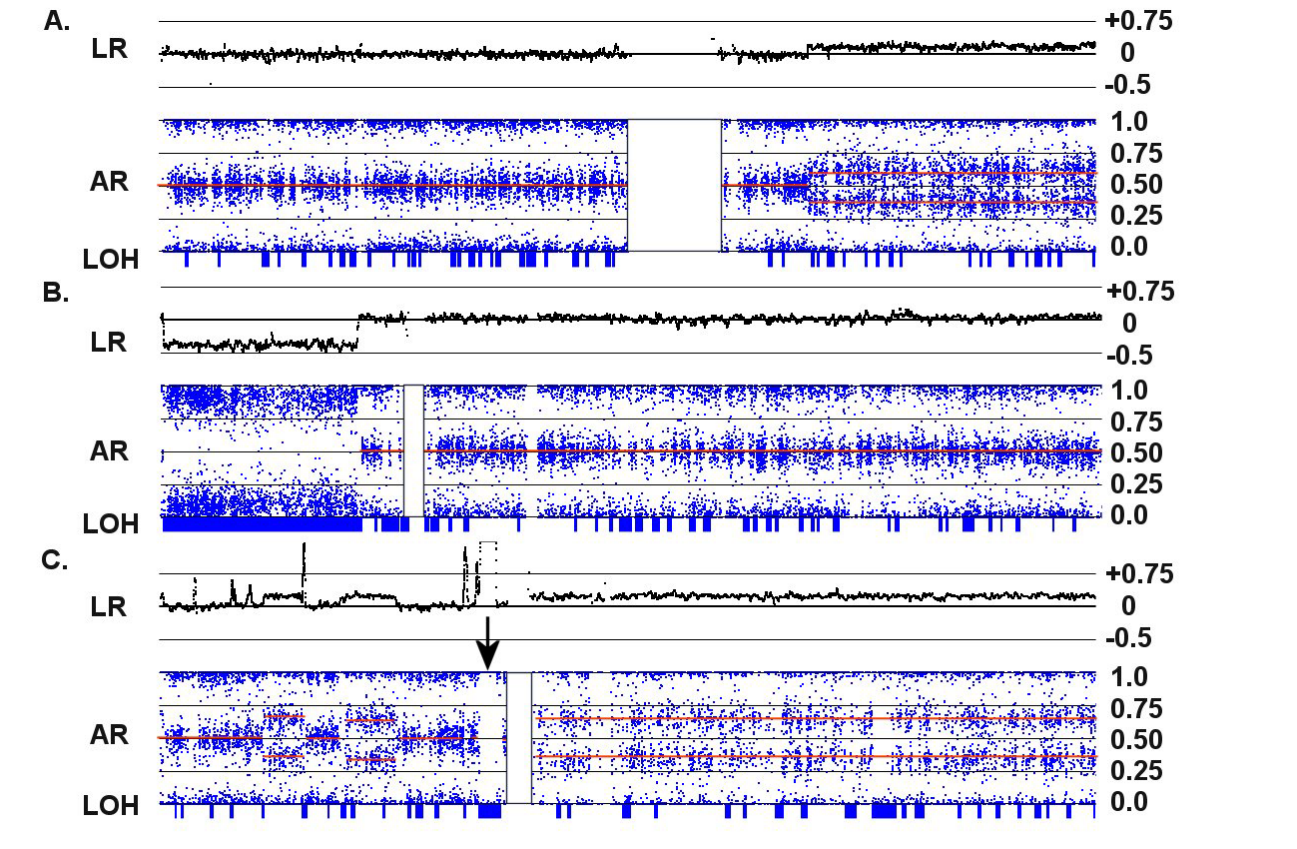
**Copy number changes in a diploid background**. Each panel shows an integrated view of log ratios (LR), allelic ratios (AR) and LOH. (A) A single copy gain in the q-arm of the disomic Chr:1 in C82 is detected by a 0.25 increase in the log ratio and a shift in the AR to a trisomy pattern (i.e., from 0.5 to 0.33 and 0.67). (B) A single copy loss in the p-arm of Chr:5 in C156 is detected by a -0.4 decrease in the log ratio and a shift in the AR to a monosomic pattern. The loss is accompanied by a collinear region of LOH (indicated by the long blue rectangle on the LOH bar). (C) A complex series of CNAs on Chr:7 in C92 is paralleled by changes in the ARs. Note that the entire q-arm is trisomic, and the strong amplification event on the p-arm adjacent to the centromere (indicated by an arrow) shifts the AR to extreme values and generates a collinear segment of LOH.

**Figure 4:**
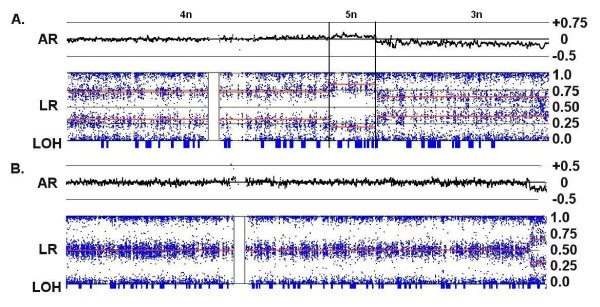
**Copy number changes in an apparent tetraploid background**. (A) Allelic ratios of 0.25 and 0.75 for Chr:10 in C72 indicate an unbalanced tetrasomy (labeled as "4n") for the p-arm of this chromosome, even though the log ratio is 0 and the putative copy number is 2. A decrease in the LR suggests a loss of chromosomal material in the q-arm ("3n"), but the region produces an AR pattern characteristic for trisomy with no collinear LOH. The center of the q-arm also displays a short segment of unbalanced pentasomy ("5n") accompanied by a small increase in the LR and allelic ratios consistent with ABBBB and AAAAB allele patterns. (B) Chromosome 2 in C82 exhibits a LR with an apparent CN of 2 and an AR consistent with normal disomy. However, the small deletion at the q-terminus produces a trisomic pattern and no LOH, revealing that the majority of the chromosome is actually a balanced tetrasomy (AABB). For both examples, the overall indication is that the baseline CN of the chromosome is 4 copies rather than 2, and both samples are largely tetraploid.

**Figure 5:**
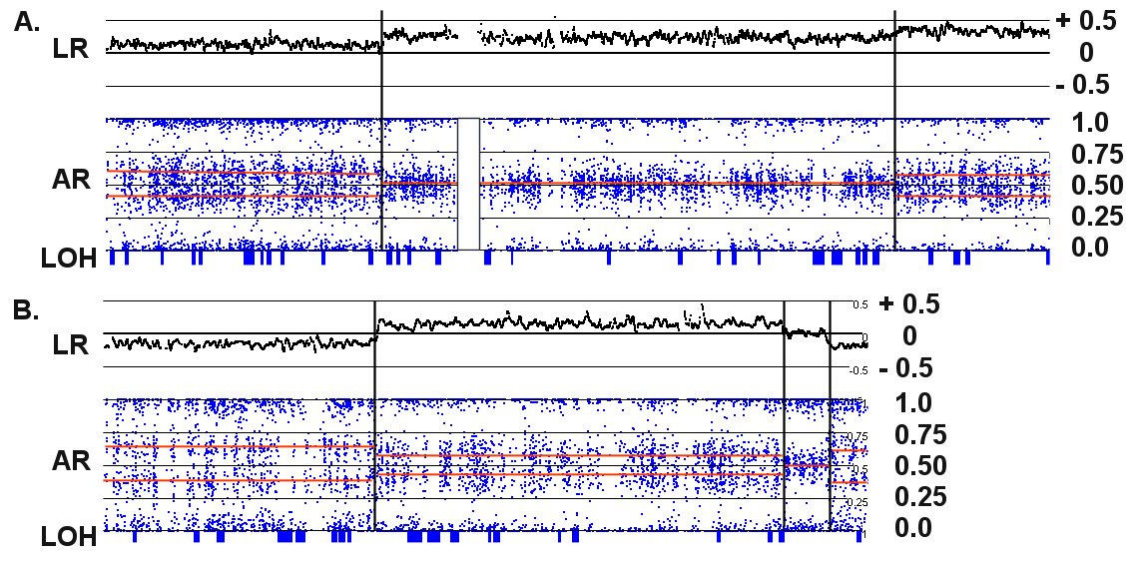
**Chromosomal CNAs demonstrating high polysomies**. (A) For chromosome 7 in C72, the LR and AR patterns are consistent with segments of CN = 5 (AAABB), 6 (AAABBB) and 7 (AAAABBB) from left to right (as partitioned by the vertical lines). The corresponding log ratios are 0.15, 0.25 and 0.35, respectively. (B) For Chr:15 in C72, the AR patterns are consistent with segments of CN = 3 (AAB), 5 (AAABB), 4 (AABB) and 3 (AAB) from left to right. The corresponding log ratios are -0.2, +0.2, 0 and -0.2, respectively. Thus, the "copy neutral" LR of 0 actually represents a CN of 4.

In addition to the log_2 _ratio (LR) of signal intensities and LOH estimations from genotypes, the allelic ratio (AR) is a useful addendum in the interpretation of copy number (CN) in tumor samples. The AR measures the contribution of the "B" allele to the total signal intensity from the two possible alleles (A or B) at each individual SNP. Since the labeling of the allele is arbitrary, the ratios form a symmetrical pattern about the value of 0.5. Allelic ratio patterns are described in a conceptual schema (Fig. 1) and are illustrated by the patterns generated from specific tumor samples (Figs. 2, 3, 4, 5). For a heterozygous, disomic chromosome, the allelic ratios should approximate to 1.0 (BB), 0.5 (AB) or 0 (AA) generating the pattern seen in Fig. 1A and Fig. 2A. The values deviate slightly from the expected ratios because of variation in individual SNP characteristics and background noise, as well as tumor heterogeneity and presence of normal cells in the tumor sample.

A heterozygous deletion on a disomic chromosome should lack the AB signals (i.e., those near 0.5), since only a single homozygous signal should be possible. In this case an AR of ~1 or ~0 would be expected since it is derived from a single copy of either the A or B allele (Fig. 2B). The AR produced from a CN of 1 tends to bleed toward the middle of the AR profile, since signal background has a greater impact on an overall signal generated by a single allele. In addition, a deletion within a disomic chromosome should be accompanied by a commensurate region of LOH. When LOH occurs at CN > 1, the allelic ratios are less affected by signal background and more closely approach the 0/1 values, as exemplified by the copy-number neutral LOH event shown in Fig. 2C.

In contrast, a region of single copy gain (CN = 3) produces a distinctly different pattern of AR that results from potential allelic combinations of AAA (0), AAB (0.33), ABB (0.67) or BBB (1.0), as seen in Fig. 2D (unless all 3 copies derive from a single parental chromosome through some combination of loss and triplication). A two-copy gain (CN = 4) may produce either of two AR patterns, depending on whether the gain is due to a duplication of both parental chromosomes or due to triplication of one of the chromosomes. The latter (unbalanced) case of tetrasomy exhibits a pattern produced by four possible allelic ratios: AAAA (0), AAAB (0.25), ABBB (0.75) or BBBB (1.0), as in the p-arm in Fig. 2E. On the other hand, balanced tetrasomy produces a pattern of AR similar to that of the normal heterozygous disomic state having possible allele combinations of AAAA (0), AABB (0.5) or BBBB (1.0).

### Integrated interpretation of CN changes using LR, AR and LOH

In a largely diploid cell, the interpretation of changes in signal ratios and allelic ratios is relatively straightforward, as illustrated in Fig. 1A; a one copy gain should increase the log ratio and produce a trisomy AR pattern, while a one copy loss should decrease the log ratio and produce a monoploid pattern with LOH. Fig. 3A depicts a case in which a gain relative to the baseline log ratio of 0 does, in fact, represent a shift in CN from 2 to 3 for the q-arm of the chromosome, with a concomitant shift in the AR from 2n to 3n. Fig. 3B illustrates the converse case, where a loss within the p-arm of a disomic chromosome produces a negative LR and an AR lacking signal typical of heterozygosity (i.e., no AR at 0.5). This loss is accompanied by LOH, as expected for a region with a heterozygous deletion.

Identification of a whole chromosome gain resulting in trisomy is generally unambiguous, since the AR displays a characteristic pattern reflecting AAB and ABB profiles (Fig. 2D) that shift away from the balanced ratio of 0.5. Note also that the AR of a balanced pentaploid (AAABB) should begin to approach the ratio seen for triploids (i.e., 3/5 vs. 2/3).

On the other hand, tetraploid states may be more difficult to assign; while unbalanced tetrasomy (AAAB) generates characteristic 0.25/0.75 ARs (as in the p-arm shown in Fig. 4A), both balanced tetrasomy and disomy produce ARs of 0, 0.5 and 1. The log ratio may not distinguish the latter two cases, since in tetraploid samples the baseline LR would be also be 0 (as in Fig. 4A and 4B). Fortunately, balanced tetrasomy can be identified in cases where subregions of gain or loss produce AR patterns that are not consistent with a baseline disomic state. For example, the q-terminus of the chromosome shown in Fig. 4B illustrates a case where the log ratio indicates a copy number loss, but this is in fact a shift from a CN of 4 to a CN of 3, rather than a heterozygous deletion on a disomic chromosome. There are three pieces of evidence that suggest that this overall pattern represents a balanced tetrasomy rather than disomy: a) the subregion containing the loss reflects an AR pattern consistent with trisomy, b) there is no region of LOH collinear with the loss, which would have occurred if the CN had been reduced from 2 to 1, and c) the magnitude of the decrease in the LR is less than expected.

More complex patterns can be characterized by combining information from both the LR and the AR, as illustrated in Fig. 5. Panel A shows a case where the deduced background state (LR = 0) is tetraploid, since LR gains of 0.15, 0.25 and 0.35 produce ARs consistent with balanced CN states of 5 (AAABB), 6 (AAABBB) and 7 (AAAABBB), respectively. In another chromosome from the same sample (Panel B), an LR loss of -0.15 produces a trisomy pattern (at the p- and q-termini) and a gain of 0.15 produces a pentasomy pattern. Furthermore, the region of LR loss is not accompanied by LOH. In this case, the small segment of "copy neutral" signal (LR = 0) must represent a region of balanced tetrasomy.

### Magnitude of LR changes in diploid and polyploid backgrounds

**Figure 6:**
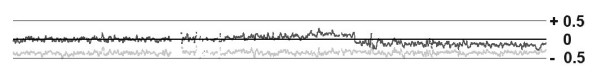
**Relative magnitude of LR changes in diploid and tetraploid backgrounds**. The lighter signal trace represents loss of the entire chromosome 10 in C172 where an LR of 0 corresponds to a CN of 2. In this case, a CN loss of 1 produces a LR of -0.4. In C72 where the background is largely tetraploid (the dark signal trace), a single copy loss (i.e., from CN of 4 to CN of 3) in the q-arm of chromosome 10 results in a decrease of the LR to only -0.15.

In a normal sample, with predominantly diploid chromosome numbers, the expectation would be that a CN of 2 corresponds with an LR of 0, since total signal at each SNP should be nearly equal to that produced by the reference set of Hap Map samples (i.e., log_2_(2/2) = 0). In an ideal milieu, a CN of 1 would produce an LR of -1 (log_2_(1/2)) and a CN of 3 would produce an LR of +0.58 (log_2_(3/2)). Empirically, these values are somewhat compressed by the effect of non-specific signal background to -0.4 and +0.3 for losses and gains, respectively. However, in a predominantly tetraploid sample, where an LR of 0 corresponds to a CN of 4, the ratios associated with losses and gains will generally be smaller than in the diploid background. The expected ideal log_2 _ratio would be -0.41 (log_2_(3/4)) and +0.32 (log_2_(5/4)) for single copy losses and gains, respectively. Empirically, it appears these values are compressed to about -0.15 and +0.1 in a tetraploid background (Fig. 6). As a result, consistent changes in LR of relatively small magnitudes are suggestive of a higher baseline CN.

### Survey of copy number changes in GBM samples

**Figure 7:**
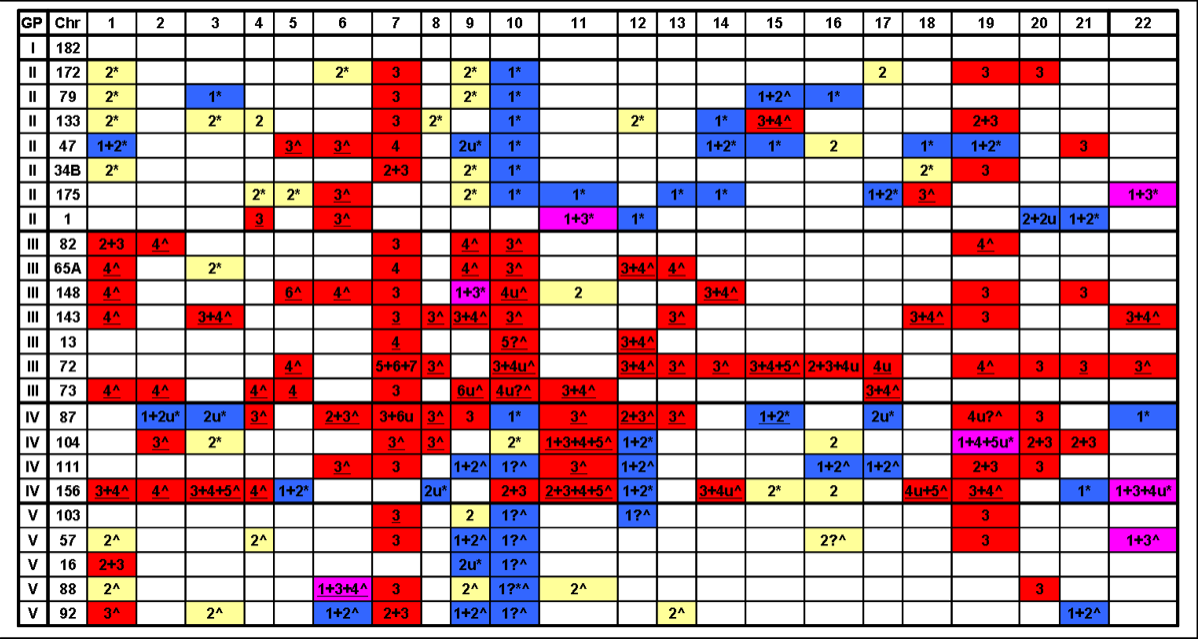
**Predominant baseline CN for assigned chromosomes of 24 tumor samples**. Codes: yellow = diploid; red = CN gain; blue = CN loss; pink = mixed gains and losses; u = unbalanced; * = LOH detected for losses; ^ = LOH not detected for losses; underline = CN under estimated by LR; ? = ambiguous AR pattern.

Combined analysis of LR and AR patterns for 24 GBM samples reveals that the majority of the chromosomes may be polysomic (Fig. 7). The baseline CN (i.e., at LR = 0) for 203 of the 528 (i.e., 24 samples × 22 chromosomes) autosomal chromosome sets can be estimated by this analysis, corresponding to approximately 38% of the chromosomes. In many cases, the baseline CN is readily apparent due to the general unbalanced pattern of the AR (as in Fig. 4A), but in other cases, a subregion of loss or gain is required to deduce the background CN state (as in Fig. 4B). The copy number of the remaining chromosomes (those with no regions of gain or loss) is actually unknown, since they may be balanced disomy (AB), balanced tetrasomy (AABB) or even higher balanced states. Of the 203 assignments, 11 are somewhat ambiguous, mostly due to the fact that the allelic ratios of unbalanced high polypsomies (i.e., AAAB or AAAAB) begin to approach the ratio of a single copy number impacted by background effects. Of the 105 chromosome sets assigned by AR patterns as CN > 2, 76 would be underestimated by the value of the LR alone.

Figure 7 also indicates the LOH state for chromosomes where an apparent one-copy loss (according to the log ratio) has occurred. The occurrence of LOH reinforces the conclusion that the baseline CN state is 2n, whereas cases where LOH is not observed tend to support the conclusion that the baseline CN state is 3n or more.

The pattern of polysomies for the 24 samples is shown for chromosomes where a putative assignment by LR, AR and LOH could be made. Figure 7 is divided into 5 categories based on gross changes in the assigned chromosomal numbers, with a basic aim of estimating the overall ploidy (i.e., inferring the CN of unassigned chromosomes) and understanding the etiology of chromosomal gains or losses for individual samples. The classification also takes into account whether the deduced CN would have been underestimated by the log ratio. The rationale for this factor is that samples with a large proportion of polysomic chromosomes would tend to reset the genome-wide log ratio baseline to a higher CN, meaning that, for the unassigned chromosomes in these samples, a LR of 0 probably represents a CN > 2.

The five categories (the number of samples in each group is indicated in parentheses) are:

I. (1) No observable change in overall chromosome CN. This group comprises one sample (C182) exhibiting an AR consistent with balanced diploidy and having no subregions of gain or loss that impacted the AR. While putatively diploid, a completely balanced tetraploidy could not be ruled out for this sample.

II. (7) Probable diploid background: The majority of the assigned chromosomes have a CN of 2 or less; the LR tends to correctly indicate chromosomal gains (e.g., an AR indicating triploidy is paralleled by a + 0.3 gain in the LR); and negative LR values tend to produce corresponding segments of LOH. Thus, the overall indication is that the sample is mostly diploid; interpretations based solely upon signal ratios are probably correct. Sample C133, in which 10 of the 22 autosomes can be definitively assigned to a baseline CN, is prototypical of Group II; five chromosomes are disomic, showing LOH at putative heterozygous deletions, and two others exhibit whole chromosome loss with complete LOH. C133 also shows two whole-chromosome gains, and substantial duplication in another chromosome. The gains in C133 are mostly reflected by log ratios in the range (~+0.3) expected for an overall CN near two for this sample. The samples in Group II appear to have approximately equal propensities to undergo either gross gains or losses, although C47 shows three whole-chromosome losses, substantial losses in 3 more chromosomes, and copy number neutral LOH for all of chromosome 9.

III. (7) Probable tetraploid background: The majority of the assignable chromosomes have CN > 2, including extensive balanced tetrasomy; putative losses do not produce corresponding segments of LOH; and the LR generally underestimates the CN, implying that most of the unassigned chromosomes have an actual CN of 4 or more. In C73, for example, 6 chromosomes exhibit complete or substantial balanced tetrasomy, and the overall evidence suggests that the unassigned chromosomes are also tetrasomic. These observations suggest that an endoreduplication event may have been the basic determinant of the chromosomal status for C73, although at least 5 chromosomes would have undergone additional large scale events. Within Group III, the CN of 45 of the 58 chromosomes assigned by AR patterns as "gains" would have been underestimated by the LR alone and interpreted as copy neutral or losses.

IV. (4) A heterogeneous mixture with many cases of both whole chromosome gains and losses, indicating a complex chronology of chromosomal aberrations. C87, in particular, shows dramatic and divergent chromosomal changes; 16 of the 22 autosomes have undergone gross gains or losses. Ten chromosomes show substantial gains, 4 show substantial losses, and 5 more have complete LOH in regions within CN of 2 or more. C156 illustrates another complex situation with many instances of whole chromosomes or large sub-regions displaying CNs of 1, 2, 3, 4 (balanced and unbalanced) and 5.

V. (5) Ambiguous: The LR tends to correctly predict the CN (implying a diploid background), although putative deletions do not produce corresponding regions of LOH. This phenomenon may represent a set of mostly diploid tumor samples with some contamination by normal tissue, which may mislead the LOH algorithm with spurious heterozygotic genotypes.

We cannot rule out the possibility that in some cases, the observed patterns are due to heterogeneity in the tumor population. Some of the AR and LR patterns can be mimicked by mixtures of substantial proportions of two subpopulations carrying different chromosomal complements. For example, chromosomes 5 and 6 of sample C47 both give trisomic patterns but negative log ratios. Since the sample appears to have a predominantly diploid background, the loss in the LR signal suggests that these two chromosomes have in fact undergone a net loss of chromosomal material. An equal mixture of cells with CN of 1 and CN of 2 for a particular chromosomal segment will generate a trisomic AR pattern; on average heterozygous SNPs will give 1:2 or 2:1 allelic ratios (AB from one subpopulation plus either A or B from the other subpopulation). In such a situation, the total signal will decrease by 25% since there will be an average of 1.5 copies of this chromosomal segment per cell rather than the normal 2 per cell in a diploid sample. In this scenario, the subpopulation losses in chromosomes 5 and 6 of sample C47 probably occurred subsequent to losses in chromosomes 1, 9, 10, 14, 15, 18 and 19 and gains in chromosome 7 and 21 since these events appear to be consistant throughout the sample.

### Re-interpretation of chromosomal alterations based on AR

Lo et al. [13] noted that many chromosomal regions of these GBM genomes showed consistent regions of loss, and the minimal regions of overlap among the effected samples was defined. In some cases, the common region of loss occurred in 2/3 of the samples. These CNAs were indicated by decreases in the LR obtained from BAC array CGH or 100 K Mapping arrays, though the authors noted that the affected regions were not necessarily accompanied by LOH, as would be expected for disomic chromosomes. Re-interpretation of the chromosomal status in light of AR and LOH patterns indicates that, in many cases, putative whole-chromosome losses are in fact trisomic, and many apparent losses actually occur against polyploid backgrounds, producing regions that are at least 2n with no concomitant LOH.

The examples below show that interpretation of CN loss based solely upon log ratios may be misleading, especially in cases of apparent whole-chromosome loss. However, the repeated occurrence of relative losses at particular regions indicates potentially interesting regions, even when particular relative losses do not lead to single-copy or zero-copy regions and LOH. In some of the examples below, multiple-copy deletions occur against polyploid backgrounds with the implication that the deletions occurred after whole chromosome duplication or even triplication.

Interpretation of polysomy is especially important for Chromosome 10, which frequently displays whole chromosome losses in GBM. Chromosome 10 carries the PTEN tumor-suppressor gene (.chr10:89287772–89390708), which has been implicated in the etiology of GBM. Strictly according to signal ratio [13], 18 of the 24 samples show whole chromosome loss of chromosome 10, C72 shows a large deletion in the q-arm, and C156 shows an extensive gain in the p-arm. However, when re-interpreted in light of the AR, only 7 samples (see Fig. 7) are clearly monosomic with strong LOH. Instead of whole chromosome losses, chromosome 10 of C65A, C143 and C82 are triploid, and C148 clearly has unbalanced tetrasomy. The putative deletion in the q-arm in C72 is, in fact, a shift in CN from 4 to 3, with the trisomic region carrying the PTEN gene (see Fig. 4A). The gain in the p-arm in C156 is consistent with a trisomic region on a balanced disomic background. Eight samples show ambiguous AR patterns consistent with either unbalanced polysomy or with whole chromosome loss, but do not show LOH. Overall, rather than a consistent pattern of chromosomal losses, definitive assignments by AR show that 5 samples have Chromosome 10 gains including the region of PTEN, four more are at least disomic at PTEN, and only seven clearly represent whole chromosome losses accompanied by LOH.

Eight of these samples show apparent losses in chromosome 14q, including 5 with whole-chromosome losses and another 3 showing apparent homozygous deletions with the minimal region of overlap at 58–78 Mb. The inference from AR patterns is that two whole-chromosome losses are, in fact, disomic and another is trisomic, while two of the sub-chromosomal deletions are single copy losses against a tetrasomic background (i.e., trisomic). The three remaining samples do appear to be monosomic at 58–78 Mbp, demonstrating LOH in this region. Similar patterns are seen for the regions of common putative losses in chromosomes 12 and 15.

The 11p region appears to show extensive losses in the LR of 7 samples. However, two of the three putative whole-chromosome losses represent trisomy according to the AR pattern. Three of the four partial chromosome losses occur against trisomic or tetrasomic backgrounds. Interestingly, in two of these three cases, 2 copies are lost from a trisomic background (C1) and 2 copies are lost from a tetrasomic background (C156) over the entire p-arm, and both produce LOH. Thus, of the 7 apparent loses, three represent trisomy without LOH and four represent losses accompanied by LOH. One consensus minimal region of 3.57–5.15 Mbp derives from a heterozygous deletion on a diploid background and another at 29.49–33.84 Mbp is defined by copy-number neutral LOH (both in sample C88).

The q-arm of chromosome 6 also shows patterns of specific regional losses, even against a background of polyploidy. Ten samples show large scale alterations in the CN of chromosome 6, including 6 samples with a baseline CN of 3, and one more with a baseline CN of 4. Six of the chromosomes show large regions of relative LR loss in the q-arm, with the common region spanning 159 Mbp to the q-term (170 Mbp). Two samples (C88, C175) show 2-copy losses (and LOH) from trisomic backgrounds in the region of 155.90–165.26 Mbp. Another sample (C182) shows a small heterozygous deletion at 162.50–162.90 against a diploid background, which then defines the minimal region.

The CDKN2A locus on chromosome 9 is the site of frequent losses with a minimal region defined by BAC analysis [2] as chr9:21698049–22584980. In this analysis, chromosome 9 was the second most frequent (after chromosome 10) to show gross losses in chromosomal material, with six cases showing losses in CN or by LOH over substantial regions (Fig 7). Five samples had homozygous deletions and 7 had heterozygous deletions at CDKN2A within a disomic background. However, another 5 samples showed whole chromosome gains for chromosome 9, including four with tetrasomic status or greater. Interestingly, 3 of these polysomic chromosomes showed multiple-copy losses specifically at the CDKN2A locus, including a 4-copy loss against a 6n background. Even with multiple-copy losses, these samples still maintain at least 2n status at CDKN2A and none of the five shows LOH. This situation implies that the deletions at CDKN2A occurred after duplication or triplication of chromosome 9. Even though at least two chromosomal copies remain at this locus, other mechanisms may be responsible for inactivating the gene [20]. The general implication is that regions of multiple-copy loss against polysomic backgrounds may highlight particularly interesting regions, analogous to homozygous deletions that remove tumor suppressor genes, even if they do not eliminate the gene or even produce LOH.

### Comparison of 500 K and BAC CGH arrays

**Figure 8:**
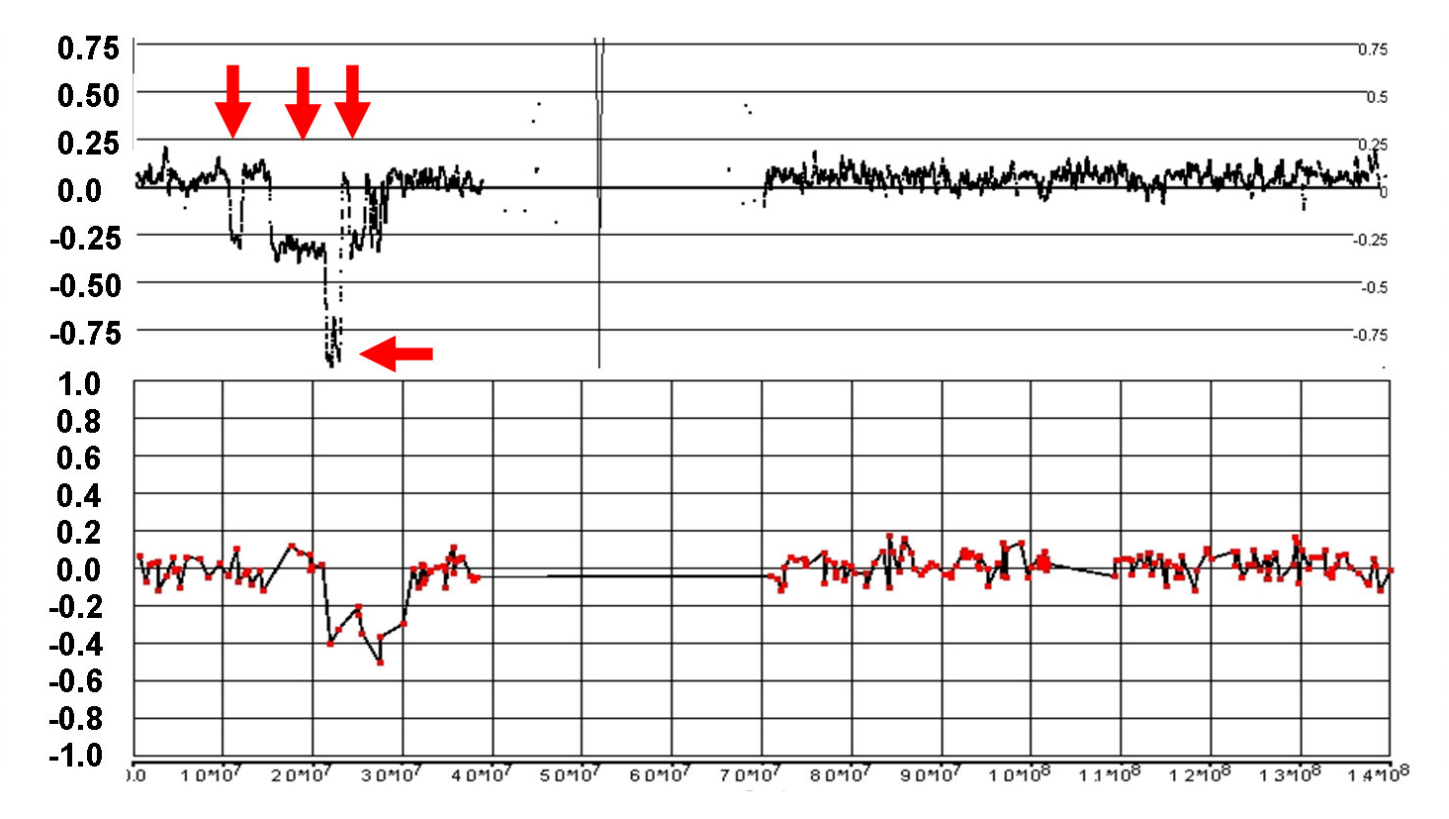
**Complex losses and gains in the q-arm of Chr:16 of sample C111**. The figure shows the LR from the 500 K (top) and 19 K BAC (bottom) arrays with the genomic scale (x-axis) indicating the SNP position or the center point of the BAC clone. The status of the CN changes is more clearly resolved with the 500 K arrays than with the BAC array, particularly for two sizeable regions of loss (between 45 Mb-54 Mb and 59 Mb – 63 Mb). Furthermore, CGH does not clearly detect a loss at 20.88 – 23.36 Mb and a copy neutral region within a deletion at 77.14 – 77.54 Mb (arrows).

The samples in this study have been previously analyzed by CGH arrays with either 6 K or 19 K BAC probes [13], with resolutions determined primarily by the average probe distance of ~500 Kb and 200 kb respectively. Affymetrix genotyping arrays with 500 K probes would be expected to have a greater resolution with an average distance of only 5.8 kb between probes. In general, the increased probe density of the 500 K arrays displays a clearer picture of many regions of complex CN variation (see Fig. 8).

**Figure 9:**
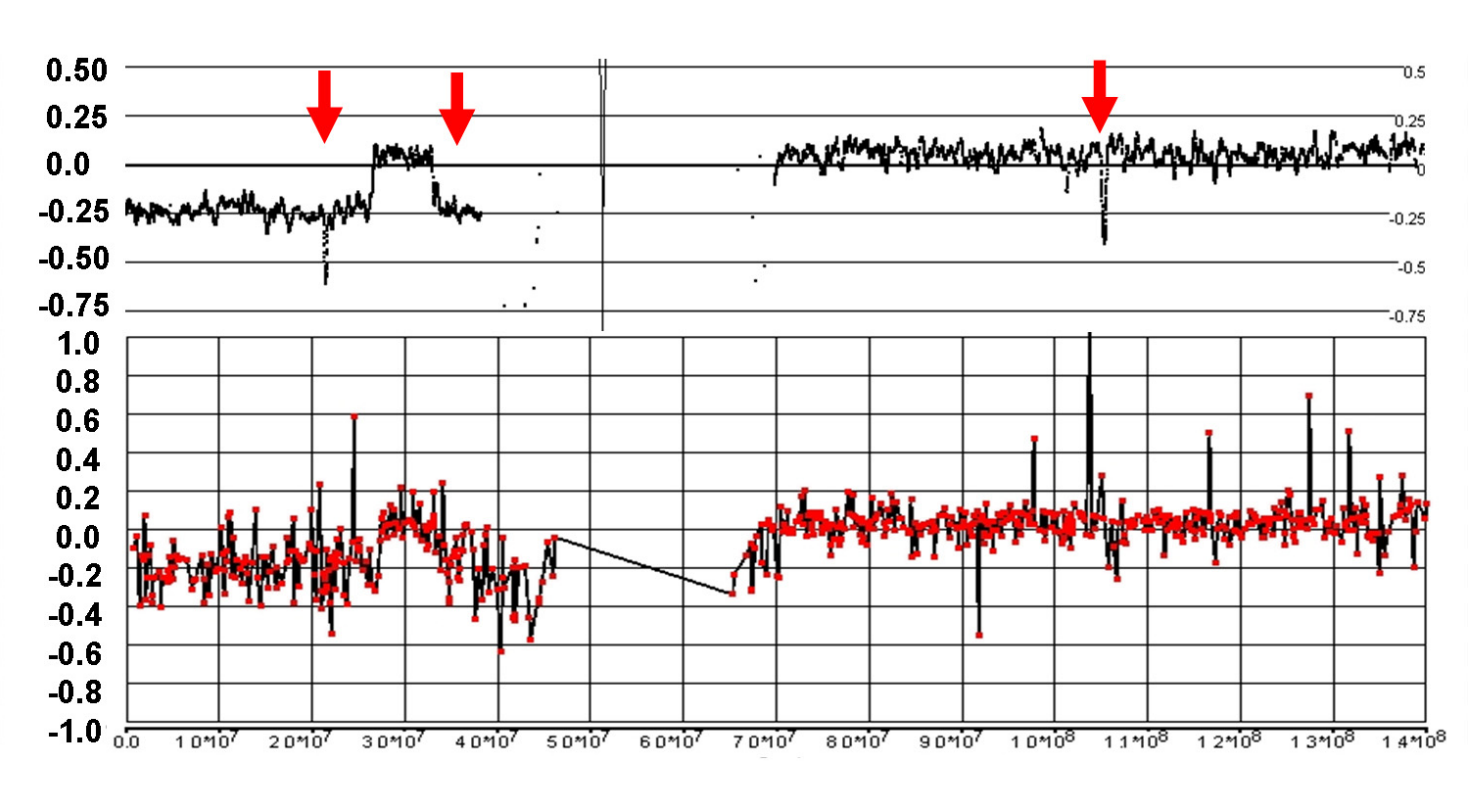
**Complex losses in the p-arm of Chr:9 of sample C79**. The 500 K array generates a detailed fine mapping of this region compared with the grosser image from the 6 K BAC array. In particular, the BAC array misses losses at 10.32 – 11.83 Mb and 14.94 – 21.07 Mb, as well as a copy neutral region from 22.90–23.76 Mb (vertical arrows). Furthermore, the 500 K mapping clearly delineates a region of homozygous deletion from 21.07 – 22.90 that is not apparent on the BAC array (horizontal arrow).

**Figure 10:**
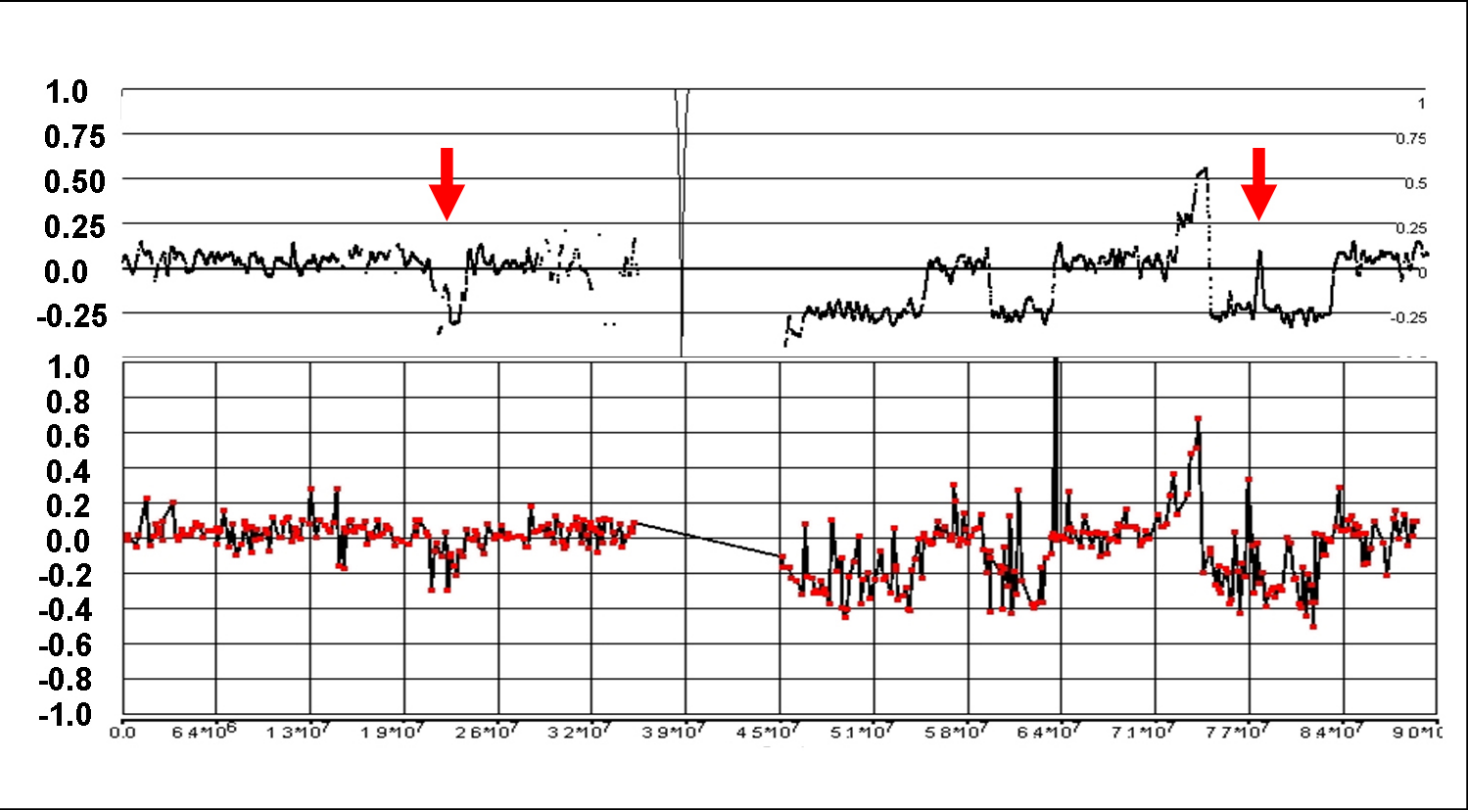
**Discrepancies between 500 K and 19 K BAC arrays for Chr:9 of sample C111**. A 5 Mb loss at 33.65 – 38.70 Mb, and two small homozygous deletions at 21.86 – 22.21 Mb and 103.42 – 103.85 Mb are apparent in the 500 K mapping, but not definitively detected by CGH.

In a detailed survey of the same 24 samples analyzed on the 500 K arrays, 422 chromosomal subregions were defined that deviate from a CN of two. There were 129 discrepancies found in CNAs that involve either gains/losses not found in the CGH analysis, or large differences (> 2 Mb) in the borders of altered regions. Of these discrepancies, 38 represented gains or losses smaller than 1 Mb that were identified only with 500 K arrays, including many in the 50 Kb range. The smaller CNAs are either not detected by any individual BAC clones (i.e., they are smaller than the resolution of the CGH) or were too inconsistent over the small region of the CNA to make a call, as shown in Fig. 9 and 10.

Another 55 of the discrepant CNAs are found in chromosomes with predominantly trisomic or tetrasomic backgrounds. Against this higher CN background, the change in the LR is relatively small (± 0.1 – 0.2) and is not detected by the CGH algorithm as CNA. The largest of the discrepancies was a loss of 46.29 Mb that affects approximately half of the p-arm of chromosome 12 in sample C13. This chromosome is otherwise tetrasomic and the loss is indicated by an LR change of only -0.1 in the 500 K signal, but the altered region is still consistent and unambiguous, and furthermore accompanied by shift to a trisomy pattern of AR on the 500 K platform.

Most of the cases of large discrepancies (> 2 Mbp) involve extensive regions of complex variation are not well resolved by CGH, but the fine structure is well mapped by the greater resolution of the 500 K array (as in the p-arm of chromosome 9 of sample C111, Fig. 9). Finally, some of the discrepancies result from highly variable signals from BAC clones in that region, producing ambiguous classification in CGH (as in Figs. 8 and 10). All of the 17 regions identified solely by CGH were rejected in the 500 K analysis as noise inherent in the sample DNA from a few of the tumors.

## Discussion

CGH and genotype mapping arrays have previously been used to detect changes in chromosomal CN in tumor samples with high resolution. These analyses, however, are in fact detecting relative changes against a background CN state that might or might not be diploid. Tetraploid cells would tend to produce a "copy neutral" LR of 0, even though naïve expectation might be that the overall signal would be twice the intensity of the reference (diploid) set with LR of +1 (log2 (4/2)). There are two major factors that adjust the LR to a value near zero, one experimental and one analytical. Primarily, only a fixed amount of DNA is assayed, in accordance with experimental protocol, without regard to the precise number of cells from which the DNA was extracted. Furthermore, the overall signal intensities are computationally normalized to the reference set to adjust for variations in experimental processes, which tends to re-adjust the overall sample signal to match the diploid state of the reference. Most algorithms that compute CN ratios make the assumption that there are the samples do not deviate significantly from diploidy, an assumption that may be frequently violated in tumors.

Lo et al. [13] previously noted, from a study of GBM tumor samples using the 100 K Mapping Array, that some chromosomal regions with an apparent CN loss (according to a decrease in the log ratio) lacked a collinear region of LOH. This discrepancy led to the suggestion that the baseline CN of these chromosomes might be greater than two, since heterozygous losses against a diploid background should always produce homozygous genotypes composed of single alleles.

It is evident from the AR and pattern of LOH that the majority of the chromosomes in many of these samples, in fact, represent trisomy or tetrasomy even though the log ratio calculations assume that the baseline chromosomal CN is 2. In most cases, the actual (higher) CN state is deduced solely from the AR pattern produced by regions of small deletions or gains that indicate a background of balanced tetrasomy (AABB). The CN assignments based on the AR largely account for the cases in which apparent deletions did not produce corresponding regions of LOH in the earlier analysis.

The presence of chromosome polyploidy forces a re-interpretation of tumor CN estimates that are based on relative signal from tumor DNA against reference samples. For example, according to log ratios, chromosome 10 appears to undergo frequent whole chromosome loss in these tumors. However, interpretation based on AR and LOH forces the conclusion that chromosomal gains may be more frequent for chromosome 10 than are losses. This is not to say that CNA in chromosome 10, and particularly at the PTEN locus, are not relevant to GBM tumorigenesis. In fact, 7 of the 24 samples do show whole-chromosome loss accompanied by LOH for chromosome 10, and two of these (C34B and C54A) carry homozygous deletions at the PTEN locus. Interestingly, 20 of the 24 samples show an unbalanced allelotype in chromosome 10 (Fig. 7), suggesting that gene dosage effects at PTEN may be important in tumorigenesis.

Several examples, particularly for the CDKN2A locus of chromosome 9, demonstrate that even apparent homozygous deletions may actually represent partial losses against a polysomic background. In these cases, the loss occurred only after whole chromosome duplication or triplication, and no general LOH is seen at that site. Still, 2- or even 4-copy losses are seen in specific regions that have previously been implicated as possible tumor suppressors. The implication is that the remaining copies of the affected genes are inactivated by another mechanism.

The 500 K and CGH-BAC array agreed overwhelmingly in determining regions with changes in CN status within a diploid background, and generally closely agreed on the breakpoints. However, the 500 K array was capable of detecting putative sub-megabase CNAs that were missed by the CGH arrays. In some cases, the signal change was detected by a single BAC clone but not in genomically neighboring clones, and was therefore ignored by the CGH calling algorithm. Many of the larger discrepancies between the platforms occurred against a background of polyploid chromosomes, which the CGH algorithm fails to call because it is not tuned to detect the relatively smaller changes in LR that are seen in this background. The 500 K array was also superior in resolving regions with complex CN aberrations.

Finally, the ability of genotype mapping arrays to detect signal from individual alleles allowed the calculation of both AR and LOH, thus providing an advantage over CGH arrays, which only detect total signal without regard to genotype. The allelic ratios operate in "genotype space", being derived from signals particular to the individual alleles; ARs are a transformation of the pattern seen by genotyping algorithms (in this case, BRLMM). The AR reflects the additional information contained in genotypes, allowing visualization of the effects of LOH and pattern shifts due to multiple copies of alleles. Here, this advantage has been leveraged to deduce that the overall CN state of most of the GBM tumor samples are higher than previously demonstrated by hybridization arrays.

## Conclusion

Prevalent polyploidy in tumor tissue has been difficult to identify by total signal on hybridization arrays due to experimental and computational normalization to a virtual diploid state; only relative changes against a baseline copy number are generally detected. Leveraging allele-specific signals in an integrated analysis allowed us to assign almost 40% of the chromosomes in GBM tumors to absolute CN states and determine that approximately half of the 24 samples surveyed appear to have general polyploidy. About 20% of the definitively assigned chromosomes had a CN >2, but three-fourths of these would have been underestimated by log signal ratio alone.

Some aberrant CN states were apparent due to unbalanced AR states (i.e., triploids), but in other cases the baseline chromosomal CN was only inferred by a combination of log ratio, allelic ratio and LOH. Since this inference may be based on deviant patterns in relatively small chromosomal regions, the high probe density and the capability of the 500 K arrays to track individual allele signals was critical in identifying regions of polysomy.

The presence of frequent polyploidy may have a strong impact on the interpretation of CNAs in tumors and particularly on expression/gene dosage studies since apparent losses may actually be only relative losses against a high CN background. Estimations of CN loss based solely upon log ratios may be misleading, especially in cases of apparent whole-chromosome loss. In the context of re-interpretation, we have identified several instances in which regions containing candidate tumor suppressors show consistently unbalanced allele states rather than losses (PTEN), or exhibit losses against a polysomic background that do not produce single- or zero-copy regions or LOH, yet apparently target a specific locus (CDKN2A).

## Abbreviations

CN: copy number; LOH: loss of heterozygosity; CNA: copy number aberration; BAC: bacterial artificial chromosome; SNP: single nucleotide polymorphism; LR: log ratio; AR: allelic ratio; GBM: glioblastoma multiforme; BRLMM: Bayesian Robust Linear Model with Mahalanobis; HMM: hidden marker model; IQR: inter-quartile range; CGH: comparative genome hybridization.

## Authors' contributions

JC and YT conceived, designed and coordinated this study. KL and PG performed data analysis. PG drafted the manuscript, and JC and YT provided critical reviews and editing. All authors read and approved the final manuscript.
